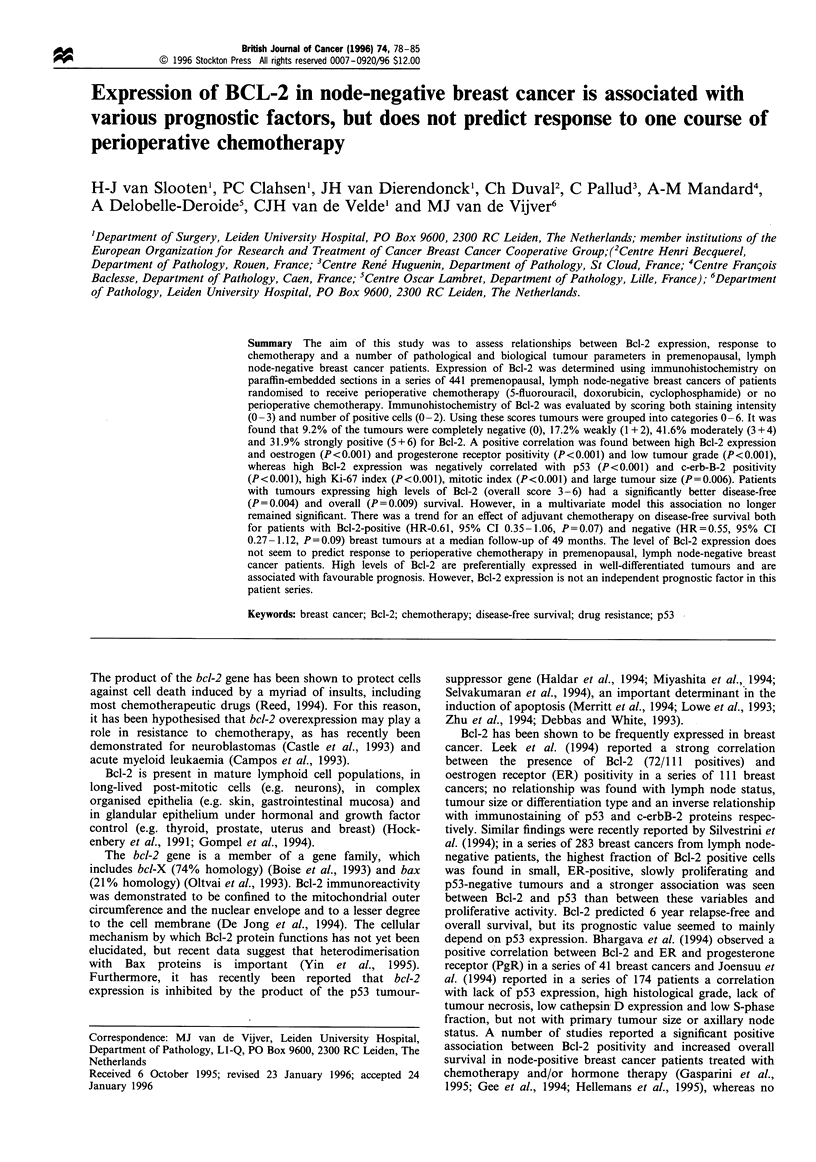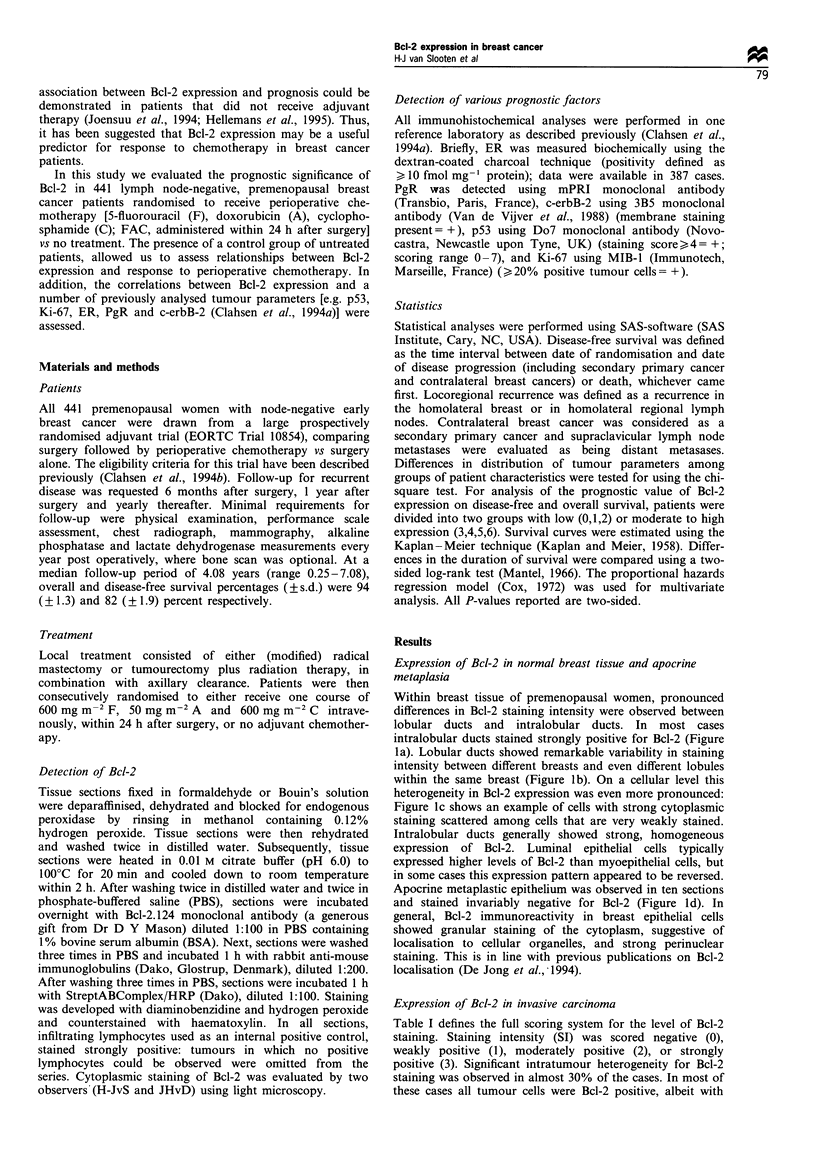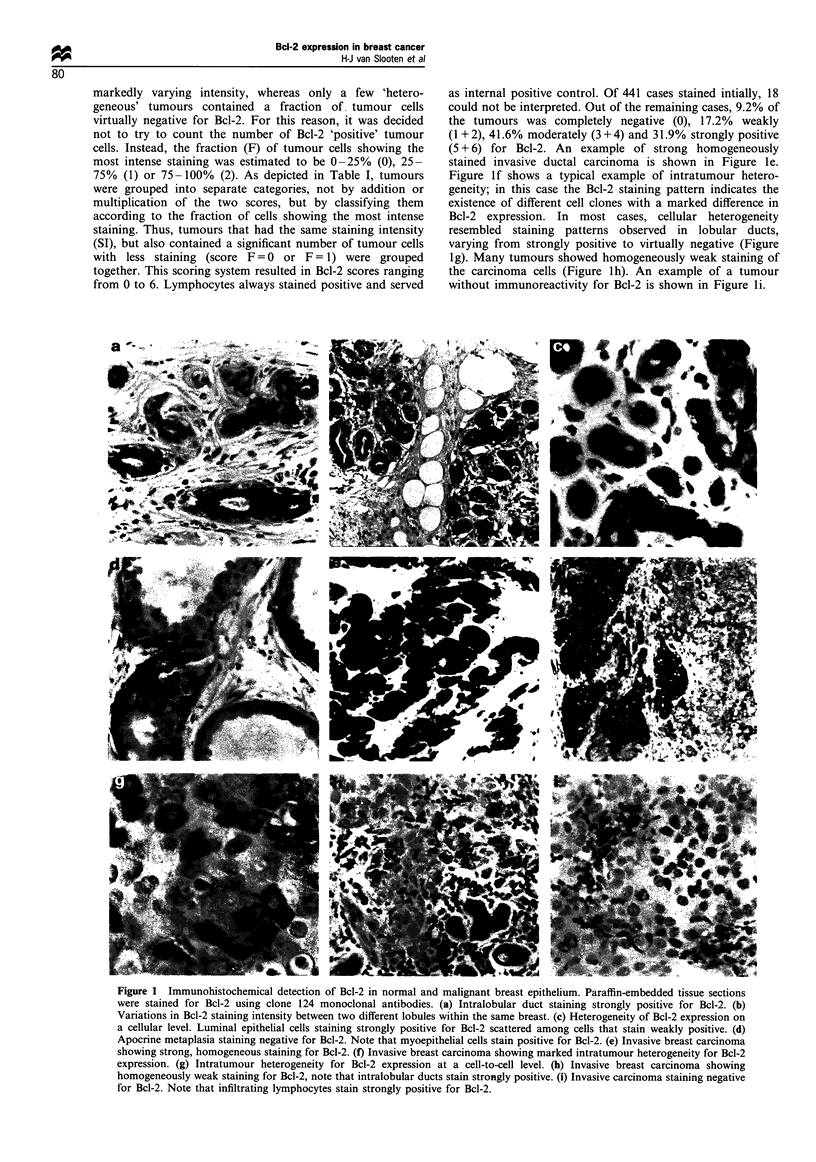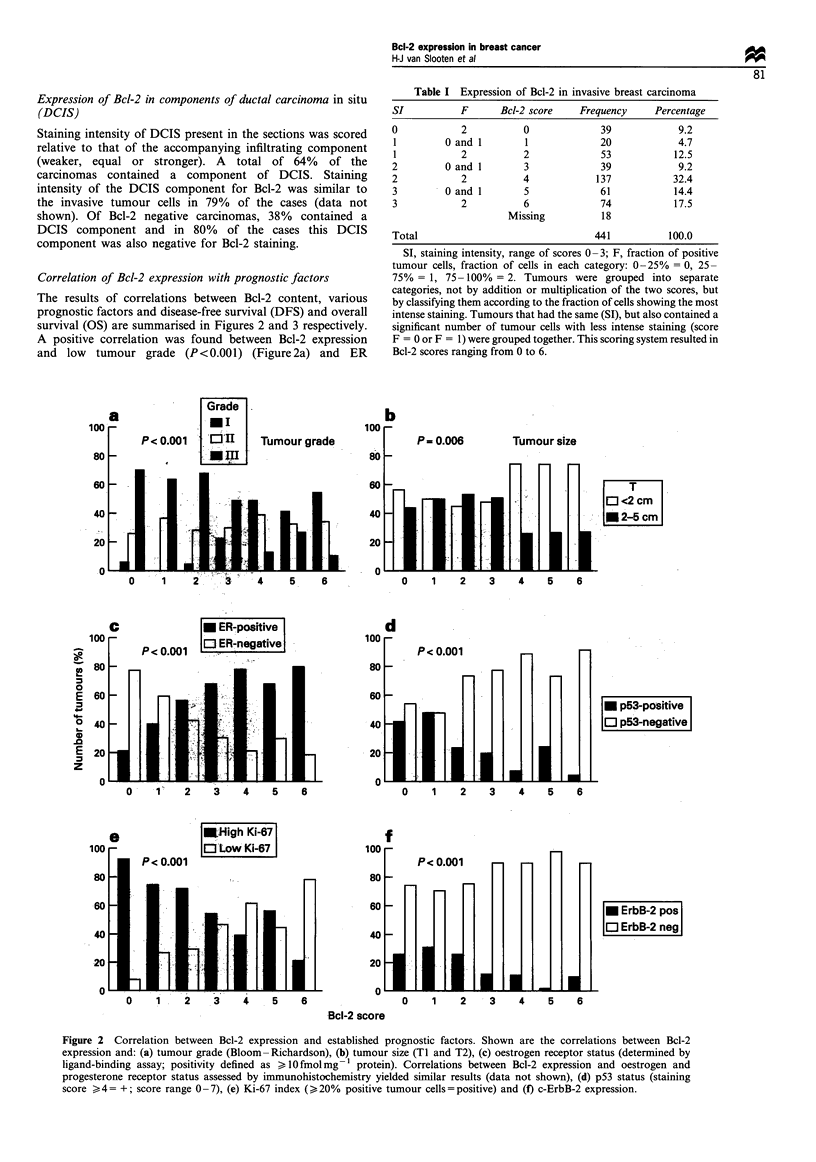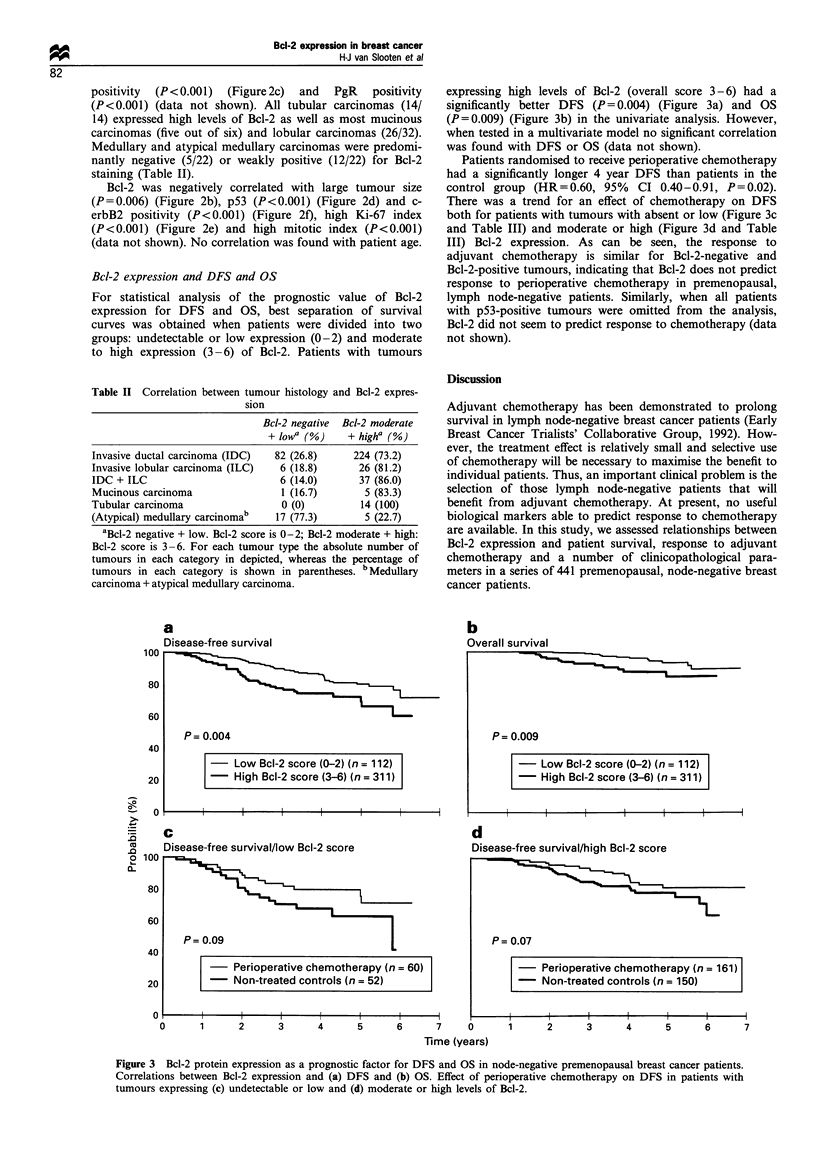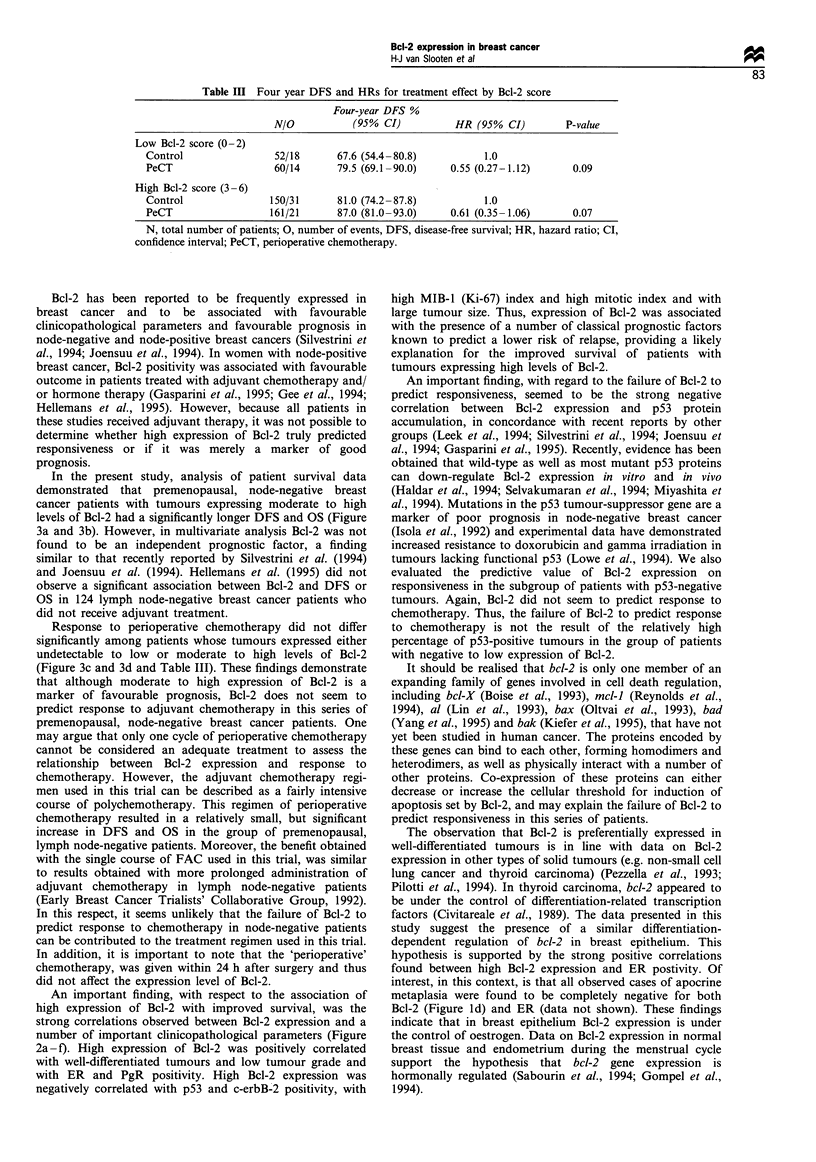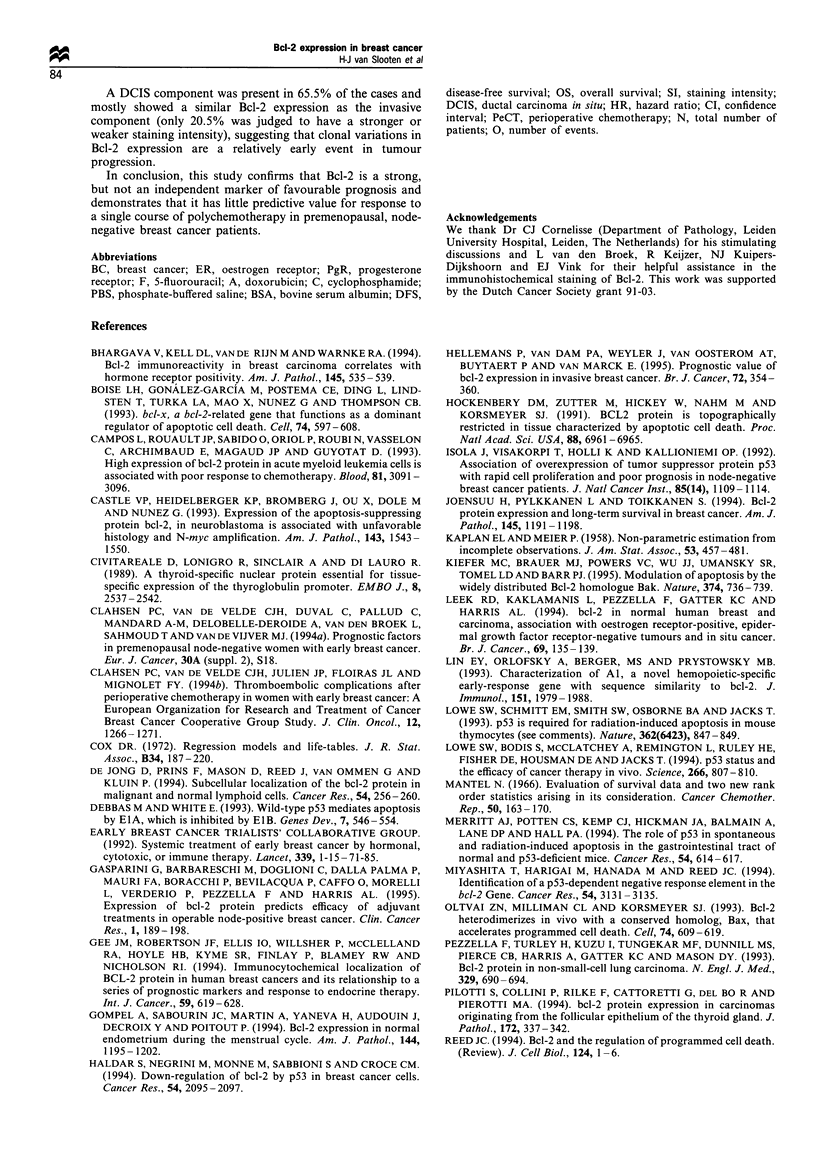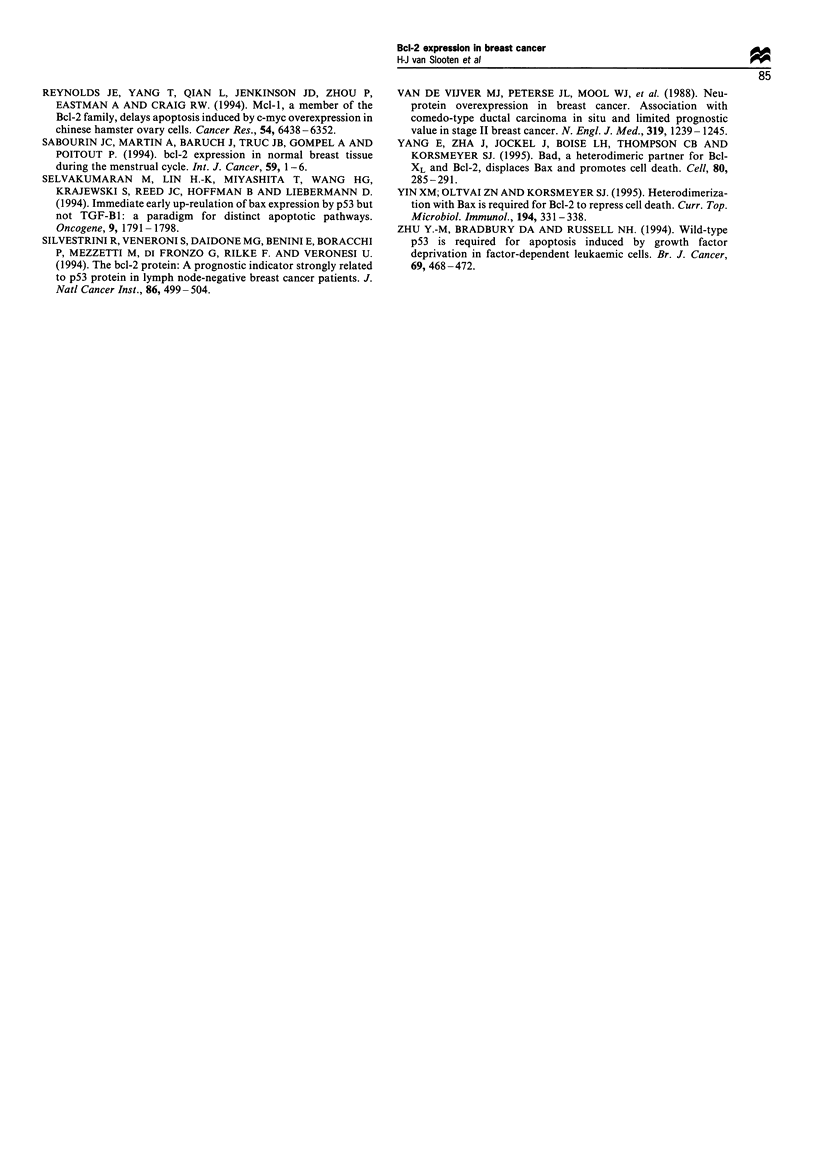# Expression of Bcl-2 in node-negative breast cancer is associated with various prognostic factors, but does not predict response to one course of perioperative chemotherapy.

**DOI:** 10.1038/bjc.1996.319

**Published:** 1996-07

**Authors:** H. J. van Slooten, P. C. Clahsen, J. H. van Dierendonck, C. Duval, C. Pallud, A. M. Mandard, A. Delobelle-Deroide, C. J. van de Velde, M. J. van de Vijver

**Affiliations:** Department of Surgery, Leiden University Hospital, The Netherlands.

## Abstract

**Images:**


British Journal of Cancer (1996) 74, 78-85
© 1996 Stockton Press All rights reserved 0007-0920/96 $12.00

### Expression of BCL-2 in node-negative breast cancer is associated with various prognostic factors, but does not predict response to one course of perioperative chemotherapy

H-J van Slooten[1], PC Clahsen[1], JH van Dierendonck[1], Ch Duval[2], C Pallud[3], A-M Mandard[4], A Delobelle-Deroide[5], CJH van de Velde[1] and MJ van de Vijver[6]

[1]*Department of Surgery, Leiden University Hospital, PO Box 9600, 2300 RC Leiden, The Netherlands; member institutions of the European Organization for Research and Treatment of Cancer Breast Cancer Cooperative Group;([2]Centre Henri Becquerel, Department of Pathology, Rouen, France; [3]Centre René Huguenin, Department of Pathology, St Cloud, France; [4]Centre François Baclesse, Department of Pathology, Caen, France; [5]Centre Oscar Lambret, Department of Pathology, Lille, France); [6]Department of Pathology, Leiden University Hospital, PO Box 9600, 2300 RC Leiden, The Netherlands.*

**Summary** The aim of this study was to assess relationships between Bcl-2 expression, response to chemotherapy and a number of pathological and biological tumour parameters in premenopausal, lymph node-negative breast cancer patients. Expression of Bcl-2 was determined using immunohistochemistry on paraffin-embedded sections in a series of 441 premenopausal, lymph node-negative breast cancers of patients randomised to receive perioperative chemotherapy (5-fluorouracil, doxorubicin, cyclophosphamide) or no perioperative chemotherapy. Immunohistochemistry of Bcl-2 was evaluated by scoring both staining intensity (0-3) and number of positive cells (0-2). Using these scores tumours were grouped into categories 0-6. It was found that 9.2% of the tumours were completely negative (0), 17.2% weakly (1+2), 41.6% moderately (3+4) and 31.9% strongly positive (5+6) for Bcl-2. A positive correlation was found between high Bcl-2 expression and oestrogen ($P<0.001$) and progesterone receptor positivity ($P<0.001$) and low tumour grade ($P<0.001$), whereas high Bcl-2 expression was negatively correlated with p53 ($P<0.001$) and c-erb-B-2 positivity ($P<0.001$), high Ki-67 index ($P<0.001$), mitotic index ($P<0.001$) and large tumour size ($P=0.006$). Patients with tumours expressing high levels of Bcl-2 (overall score 3-6) had a significantly better disease-free ($P=0.004$) and overall ($P=0.009$) survival. However, in a multivariate model this association no longer remained significant. There was a trend for an effect of adjuvant chemotherapy on disease-free survival both for patients with Bcl-2-positive (HR-0.61, 95% CI 0.35-1.06, $P=0.07$) and negative (HR=0.55, 95% CI 0.27-1.12, $P=0.09$) breast tumours at a median follow-up of 49 months. The level of Bcl-2 expression does not seem to predict response to perioperative chemotherapy in premenopausal, lymph node-negative breast cancer patients. High levels of Bcl-2 are preferentially expressed in well-differentiated tumours and are associated with favourable prognosis. However, Bcl-2 expression is not an independent prognostic factor in this patient series.

**Keywords:** breast cancer; Bcl-2; chemotherapy; disease-free survival; drug resistance; p53

The product of the *bcl-2* gene has been shown to protect cells against cell death induced by a myriad of insults, including most chemotherapeutic drugs (Reed, 1994). For this reason, it has been hypothesised that *bcl-2* overexpression may play a role in resistance to chemotherapy, as has recently been demonstrated for neuroblastomas (Castle *et al.*, 1993) and acute myeloid leukaemia (Campos *et al.*, 1993).

Bcl-2 is present in mature lymphoid cell populations, in long-lived post-mitotic cells (e.g. neurons), in complex organised epithelia (e.g. skin, gastrointestinal mucosa) and in glandular epithelium under hormonal and growth factor control (e.g. thyroid, prostate, uterus and breast) (Hockenbery *et al.*, 1991; Gompel *et al.*, 1994).

The *bcl-2* gene is a member of a gene family, which includes *bcl*-X (74% homology) (Boise *et al.*, 1993) and *bax* (21% homology) (Oltvai *et al.*, 1993). Bcl-2 immunoreactivity was demonstrated to be confined to the mitochondrial outer circumference and the nuclear envelope and to a lesser degree to the cell membrane (De Jong *et al.*, 1994). The cellular mechanism by which Bcl-2 protein functions has not yet been elucidated, but recent data suggest that heterodimerisation with Bax proteins is important (Yin *et al.*, 1995). Furthermore, it has recently been reported that *bcl-2* expression is inhibited by the product of the p53 tumour-suppressor gene (Haldar *et al.*, 1994; Miyashita *et al.*, 1994; Selvakumaran *et al.*, 1994), an important determinant in the induction of apoptosis (Merritt *et al.*, 1994; Lowe *et al.*, 1993; Zhu *et al.*, 1994; Debbas and White, 1993).

Bcl-2 has been shown to be frequently expressed in breast cancer. Leek *et al.* (1994) reported a strong correlation between the presence of Bcl-2 (72/111 positives) and oestrogen receptor (ER) positivity in a series of 111 breast cancers; no relationship was found with lymph node status, tumour size or differentiation type and an inverse relationship with immunostaining of p53 and c-erbB-2 proteins respectively. Similar findings were recently reported by Silvestrini *et al.* (1994); in a series of 283 breast cancers from lymph node-negative patients, the highest fraction of Bcl-2 positive cells was found in small, ER-positive, slowly proliferating and p53-negative tumours and a stronger association was seen between Bcl-2 and p53 than between these variables and proliferative activity. Bcl-2 predicted 6 year relapse-free and overall survival, but its prognostic value seemed to mainly depend on p53 expression. Bhargava *et al.* (1994) observed a positive correlation between Bcl-2 and ER and progesterone receptor (PgR) in a series of 41 breast cancers and Joensuu *et al.* (1994) reported in a series of 174 patients a correlation with lack of p53 expression, high histological grade, lack of tumour necrosis, low cathepsin D expression and low S-phase fraction, but not with primary tumour size or axillary node status. A number of studies reported a significant positive association between Bcl-2 positivity and increased overall survival in node-positive breast cancer patients treated with chemotherapy and/or hormone therapy (Gasparini *et al.*, 1995; Gee *et al.*, 1994; Hellemans *et al.*, 1995), whereas no

Correspondence: MJ van de Vijver, Leiden University Hospital, Department of Pathology, L1-Q, PO Box 9600, 2300 RC Leiden, The Netherlands
Received 6 October 1995; revised 23 January 1996; accepted 24 January 1996

association between Bcl-2 expression and prognosis could be demonstrated in patients that did not receive adjuvant therapy (Joensuu *et al.*, 1994; Hellemans *et al.*, 1995). Thus, it has been suggested that Bcl-2 expression may be a useful predictor for response to chemotherapy in breast cancer patients.

In this study we evaluated the prognostic significance of Bcl-2 in 441 lymph node-negative, premenopausal breast cancer patients randomised to receive perioperative chemotherapy [5-fluorouracil (F), doxorubicin (A), cyclophosphamide (C); FAC, administered within 24 h after surgery] *vs* no treatment. The presence of a control group of untreated patients, allowed us to assess relationships between Bcl-2 expression and response to perioperative chemotherapy. In addition, the correlations between Bcl-2 expression and a number of previously analysed tumour parameters [e.g. p53, Ki-67, ER, PgR and c-erbB-2 (Clahsen *et al.*, 1994*a*)] were assessed.

### Materials and methods

*Patients*

All 441 premenopausal women with node-negative early breast cancer were drawn from a large prospectively randomised adjuvant trial (EORTC Trial 10854), comparing surgery followed by perioperative chemotherapy *vs* surgery alone. The eligibility criteria for this trial have been described previously (Clahsen *et al.*, 1994*b*). Follow-up for recurrent disease was requested 6 months after surgery, 1 year after surgery and yearly thereafter. Minimal requirements for follow-up were physical examination, performance scale assessment, chest radiograph, mammography, alkaline phosphatase and lactate dehydrogenase measurements every year post operatively, where bone scan was optional. At a median follow-up period of 4.08 years (range 0.25–7.08), overall and disease-free survival percentages ($\pm$s.d.) were 94 ($\pm$1.3) and 82 ($\pm$1.9) percent respectively.

*Treatment*

Local treatment consisted of either (modified) radical mastectomy or tumourectomy plus radiation therapy, in combination with axillary clearance. Patients were then consecutively randomised to either receive one course of 600 mg $m^{-2}$ F, 50 mg $m^{-2}$ A and 600 mg $m^{-2}$ C intravenously, within 24 h after surgery, or no adjuvant chemotherapy.

*Detection of Bcl-2*

Tissue sections fixed in formaldehyde or Bouin's solution were deparaffinised, dehydrated and blocked for endogenous peroxidase by rinsing in methanol containing 0.12% hydrogen peroxide. Tissue sections were then rehydrated and washed twice in distilled water. Subsequently, tissue sections were heated in 0.01 M citrate buffer (pH 6.0) to 100°C for 20 min and cooled down to room temperature within 2 h. After washing twice in distilled water and twice in phosphate-buffered saline (PBS), sections were incubated overnight with Bcl-2.124 monoclonal antibody (a generous gift from Dr D Y Mason) diluted 1:100 in PBS containing 1% bovine serum albumin (BSA). Next, sections were washed three times in PBS and incubated 1 h with rabbit anti-mouse immunoglobulins (Dako, Glostrup, Denmark), diluted 1:200. After washing three times in PBS, sections were incubated 1 h with StreptABComplex/HRP (Dako), diluted 1:100. Staining was developed with diaminobenzidine and hydrogen peroxide and counterstained with haematoxylin. In all sections, infiltrating lymphocytes used as an internal positive control, stained strongly positive: tumours in which no positive lymphocytes could be observed were omitted from the series. Cytoplasmic staining of Bcl-2 was evaluated by two observers (H-JvS and JHvD) using light microscopy.

*Detection of various prognostic factors*

All immunohistochemical analyses were performed in one reference laboratory as described previously (Clahsen *et al.*, 1994*a*). Briefly, ER was measured biochemically using the dextran-coated charcoal technique (positivity defined as $\geqslant$10 fmol $mg^{-1}$ protein); data were available in 387 cases. PgR was detected using mPRI monoclonal antibody (Transbio, Paris, France), c-erbB-2 using 3B5 monoclonal antibody (Van de Vijver *et al.*, 1988) (membrane staining present = +), p53 using Do7 monoclonal antibody (Novocastra, Newcastle upon Tyne, UK) (staining score$\geqslant$4 = +; scoring range 0–7), and Ki-67 using MIB-1 (Immunotech, Marseille, France) ($\geqslant$20% positive tumour cells = +).

*Statistics*

Statistical analyses were performed using SAS-software (SAS Institute, Cary, NC, USA). Disease-free survival was defined as the time interval between date of randomisation and date of disease progression (including secondary primary cancer and contralateral breast cancers) or death, whichever came first. Locoregional recurrence was defined as a recurrence in the homolateral breast or in homolateral regional lymph nodes. Contralateral breast cancer was considered as a secondary primary cancer and supraclavicular lymph node metastases were evaluated as being distant metasases. Differences in distribution of tumour parameters among groups of patient characteristics were tested for using the chi-square test. For analysis of the prognostic value of Bcl-2 expression on disease-free and overall survival, patients were divided into two groups with low (0,1,2) or moderate to high expression (3,4,5,6). Survival curves were estimated using the Kaplan–Meier technique (Kaplan and Meier, 1958). Differences in the duration of survival were compared using a two-sided log-rank test (Mantel, 1966). The proportional hazards regression model (Cox, 1972) was used for multivariate analysis. All *P*-values reported are two-sided.

### Results

*Expression of Bcl-2 in normal breast tissue and apocrine metaplasia*

Within breast tissue of premenopausal women, pronounced differences in Bcl-2 staining intensity were observed between lobular ducts and intralobular ducts. In most cases intralobular ducts stained strongly positive for Bcl-2 (Figure 1a). Lobular ducts showed remarkable variability in staining intensity between different breasts and even different lobules within the same breast (Figure 1b). On a cellular level this heterogeneity in Bcl-2 expression was even more pronounced: Figure 1c shows an example of cells with strong cytoplasmic staining scattered among cells that are very weakly stained. Intralobular ducts generally showed strong, homogeneous expression of Bcl-2. Luminal epithelial cells typically expressed higher levels of Bcl-2 than myoepithelial cells, but in some cases this expression pattern appeared to be reversed. Apocrine metaplastic epithelium was observed in ten sections and stained invariably negative for Bcl-2 (Figure 1d). In general, Bcl-2 immunoreactivity in breast epithelial cells showed granular staining of the cytoplasm, suggestive of localisation to cellular organelles, and strong perinuclear staining. This is in line with previous publications on Bcl-2 localisation (De Jong *et al.*, 1994).

*Expression of Bcl-2 in invasive carcinoma*

Table I defines the full scoring system for the level of Bcl-2 staining. Staining intensity (SI) was scored negative (0), weakly positive (1), moderately positive (2), or strongly positive (3). Significant intratumour heterogeneity for Bcl-2 staining was observed in almost 30% of the cases. In most of these cases all tumour cells were Bcl-2 positive, albeit with markedly varying intensity, whereas only a few 'heterogeneous' tumours contained a fraction of tumour cells virtually negative for Bcl-2. For this reason, it was decided not to try to count the number of Bcl-2 'positive' tumour cells. Instead, the fraction (F) of tumour cells showing the most intense staining was estimated to be 0–25% (0), 25–75% (1) or 75–100% (2). As depicted in Table I, tumours were grouped into separate categories, not by addition or multiplication of the two scores, but by classifying them according to the fraction of cells showing the most intense staining. Thus, tumours that had the same staining intensity (SI), but also contained a significant number of tumour cells with less staining (score F=0 or F=1) were grouped together. This scoring system resulted in Bcl-2 scores ranging from 0 to 6. Lymphocytes always stained positive and served as internal positive control. Of 441 cases stained intially, 18 could not be interpreted. Out of the remaining cases, 9.2% of the tumours was completely negative (0), 17.2% weakly (1+2), 41.6% moderately (3+4) and 31.9% strongly positive (5+6) for Bcl-2. An example of strong homogeneously stained invasive ductal carcinoma is shown in Figure 1e. Figure 1f shows a typical example of intratumour heterogeneity; in this case the Bcl-2 staining pattern indicates the existence of different cell clones with a marked difference in Bcl-2 expression. In most cases, cellular heterogeneity resembled staining patterns observed in lobular ducts, varying from strongly positive to virtually negative (Figure 1g). Many tumours showed homogeneously weak staining of the carcinoma cells (Figure 1h). An example of a tumour without immunoreactivity for Bcl-2 is shown in Figure 1i.

**Figure 1** Immunohistochemical detection of Bcl-2 in normal and malignant breast epithelium. Paraffin-embedded tissue sections were stained for Bcl-2 using clone 124 monoclonal antibodies. (**a**) Intralobular duct staining strongly positive for Bcl-2. (**b**) Variations in Bcl-2 staining intensity between two different lobules within the same breast. (**c**) Heterogeneity of Bcl-2 expression on a cellular level. Luminal epithelial cells staining strongly positive for Bcl-2 scattered among cells that stain weakly positive. (**d**) Apocrine metaplasia staining negative for Bcl-2. Note that myoepithelial cells stain positive for Bcl-2. (**e**) Invasive breast carcinoma showing strong, homogeneous staining for Bcl-2. (**f**) Invasive breast carcinoma showing marked intratumour heterogeneity for Bcl-2 expression. (**g**) Intratumour heterogeneity for Bcl-2 expression at a cell-to-cell level. (**h**) Invasive breast carcinoma showing homogeneously weak staining for Bcl-2, note that intralobular ducts stain strongly positive. (**i**) Invasive carcinoma staining negative for Bcl-2. Note that infiltrating lymphocytes stain strongly positive for Bcl-2.

*Expression of Bcl-2 in components of ductal carcinoma* in situ *(DCIS)*

Staining intensity of DCIS present in the sections was scored relative to that of the accompanying infiltrating component (weaker, equal or stronger). A total of 64% of the carcinomas contained a component of DCIS. Staining intensity of the DCIS component for Bcl-2 was similar to the invasive tumour cells in 79% of the cases (data not shown). Of Bcl-2 negative carcinomas, 38% contained a DCIS component and in 80% of the cases this DCIS component was also negative for Bcl-2 staining.

*Correlation of Bcl-2 expression with prognostic factors*

The results of correlations between Bcl-2 content, various prognostic factors and disease-free survival (DFS) and overall survival (OS) are summarised in Figures 2 and 3 respectively. A positive correlation was found between Bcl-2 expression and low tumour grade ($P<0.001$) (Figure 2a) and ER

**Table I** Expression of Bcl-2 in invasive breast carcinoma

| *SI* | *F* | *Bcl-2 score* | *Frequency* | *Percentage* |
|---|---|---|---|---|
| 0 | 2 | 0 | 39 | 9.2 |
| 1 | 0 and 1 | 1 | 20 | 4.7 |
| 1 | 2 | 2 | 53 | 12.5 |
| 2 | 0 and 1 | 3 | 39 | 9.2 |
| 2 | 2 | 4 | 137 | 32.4 |
| 3 | 0 and 1 | 5 | 61 | 14.4 |
| 3 | 2 | 6 | 74 | 17.5 |
| | | Missing | 18 | |
| Total | | | 441 | 100.0 |

SI, staining intensity, range of scores 0–3; F, fraction of positive tumour cells, fraction of cells in each category: 0–25% = 0, 25–75% = 1, 75–100% = 2. Tumours were grouped into separate categories, not by addition or multiplication of the two scores, but by classifying them according to the fraction of cells showing the most intense staining. Tumours that had the same (SI), but also contained a significant number of tumour cells with less intense staining (score F = 0 or F = 1) were grouped together. This scoring system resulted in Bcl-2 scores ranging from 0 to 6.

**Figure 2** Correlation between Bcl-2 expression and established prognostic factors. Shown are the correlations between Bcl-2 expression and: (**a**) tumour grade (Bloom–Richardson), (**b**) tumour size (T1 and T2), (**c**) oestrogen receptor status (determined by ligand-binding assay; positivity defined as $\geqslant 10\,\mathrm{fmol\,mg^{-1}}$ protein). Correlations between Bcl-2 expression and oestrogen and progesterone receptor status assessed by immunohistochemistry yielded similar results (data not shown), (**d**) p53 status (staining score $\geqslant 4 = +$; score range 0–7), (**e**) Ki-67 index ($\geqslant$20% positive tumour cells = positive) and (**f**) c-ErbB-2 expression.

positivity ($P<0.001$) (Figure 2c) and PgR positivity ($P<0.001$) (data not shown). All tubular carcinomas (14/14) expressed high levels of Bcl-2 as well as most mucinous carcinomas (five out of six) and lobular carcinomas (26/32). Medullary and atypical medullary carcinomas were predominantly negative (5/22) or weakly positive (12/22) for Bcl-2 staining (Table II).

Bcl-2 was negatively correlated with large tumour size ($P=0.006$) (Figure 2b), p53 ($P<0.001$) (Figure 2d) and c-erbB2 positivity ($P<0.001$) (Figure 2f), high Ki-67 index ($P<0.001$) (Figure 2e) and high mitotic index ($P<0.001$) (data not shown). No correlation was found with patient age.

### *Bcl-2 expression and DFS and OS*

For statistical analysis of the prognostic value of Bcl-2 expression for DFS and OS, best separation of survival curves was obtained when patients were divided into two groups: undetectable or low expression (0–2) and moderate to high expression (3–6) of Bcl-2. Patients with tumours expressing high levels of Bcl-2 (overall score 3–6) had a significantly better DFS ($P=0.004$) (Figure 3a) and OS ($P=0.009$) (Figure 3b) in the univariate analysis. However, when tested in a multivariate model no significant correlation was found with DFS or OS (data not shown).

Patients randomised to receive perioperative chemotherapy had a significantly longer 4 year DFS than patients in the control group (HR=0.60, 95% CI 0.40–0.91, $P=0.02$). There was a trend for an effect of chemotherapy on DFS both for patients with tumours with absent or low (Figure 3c and Table III) and moderate or high (Figure 3d and Table III) Bcl-2 expression. As can be seen, the response to adjuvant chemotherapy is similar for Bcl-2-negative and Bcl-2-positive tumours, indicating that Bcl-2 does not predict response to perioperative chemotherapy in premenopausal, lymph node-negative patients. Similarly, when all patients with p53-positive tumours were omitted from the analysis, Bcl-2 did not seem to predict response to chemotherapy (data not shown).

**Table II** Correlation between tumour histology and Bcl-2 expression

| | *Bcl-2 negative + low[a] (%)* | *Bcl-2 moderate + high[a] (%)* |
|---|---|---|
| Invasive ductal carcinoma (IDC) | 82 (26.8) | 224 (73.2) |
| Invasive lobular carcinoma (ILC) | 6 (18.8) | 26 (81.2) |
| IDC + ILC | 6 (14.0) | 37 (86.0) |
| Mucinous carcinoma | 1 (16.7) | 5 (83.3) |
| Tubular carcinoma | 0 (0) | 14 (100) |
| (Atypical) medullary carcinoma[b] | 17 (77.3) | 5 (22.7) |

[a]Bcl-2 negative + low. Bcl-2 score is 0–2; Bcl-2 moderate + high: Bcl-2 score is 3–6. For each tumour type the absolute number of tumours in each category in depicted, whereas the percentage of tumours in each category is shown in parentheses. [b]Medullary carcinoma + atypical medullary carcinoma.

### Discussion

Adjuvant chemotherapy has been demonstrated to prolong survival in lymph node-negative breast cancer patients (Early Breast Cancer Trialists' Collaborative Group, 1992). However, the treatment effect is relatively small and selective use of chemotherapy will be necessary to maximise the benefit to individual patients. Thus, an important clinical problem is the selection of those lymph node-negative patients that will benefit from adjuvant chemotherapy. At present, no useful biological markers able to predict response to chemotherapy are available. In this study, we assessed relationships between Bcl-2 expression and patient survival, response to adjuvant chemotherapy and a number of clinicopathological parameters in a series of 441 premenopausal, node-negative breast cancer patients.

**Figure 3** Bcl-2 protein expression as a prognostic factor for DFS and OS in node-negative premenopausal breast cancer patients. Correlations between Bcl-2 expression and (**a**) DFS and (**b**) OS. Effect of perioperative chemotherapy on DFS in patients with tumours expressing (**c**) undetectable or low and (**d**) moderate or high levels of Bcl-2.

**Table III** Four year DFS and HRs for treatment effect by Bcl-2 score

| | *N/O* | *Four-year DFS % (95% CI)* | *HR (95% CI)* | P-*value* |
|---|---|---|---|---|
| Low Bcl-2 score (0–2) | | | | |
| Control | 52/18 | 67.6 (54.4–80.8) | 1.0 | |
| PeCT | 60/14 | 79.5 (69.1–90.0) | 0.55 (0.27–1.12) | 0.09 |
| High Bcl-2 score (3–6) | | | | |
| Control | 150/31 | 81.0 (74.2–87.8) | 1.0 | |
| PeCT | 161/21 | 87.0 (81.0–93.0) | 0.61 (0.35–1.06) | 0.07 |

N, total number of patients; O, number of events, DFS, disease-free survival; HR, hazard ratio; CI, confidence interval; PeCT, perioperative chemotherapy.

Bcl-2 has been reported to be frequently expressed in breast cancer and to be associated with favourable clinicopathological parameters and favourable prognosis in node-negative and node-positive breast cancers (Silvestrini *et al.*, 1994; Joensuu *et al.*, 1994). In women with node-positive breast cancer, Bcl-2 positivity was associated with favourable outcome in patients treated with adjuvant chemotherapy and/or hormone therapy (Gasparini *et al.*, 1995; Gee *et al.*, 1994; Hellemans *et al.*, 1995). However, because all patients in these studies received adjuvant therapy, it was not possible to determine whether high expression of Bcl-2 truly predicted responsiveness or if it was merely a marker of good prognosis.

In the present study, analysis of patient survival data demonstrated that premenopausal, node-negative breast cancer patients with tumours expressing moderate to high levels of Bcl-2 had a significantly longer DFS and OS (Figure 3a and 3b). However, in multivariate analysis Bcl-2 was not found to be an independent prognostic factor, a finding similar to that recently reported by Silvestrini *et al.* (1994) and Joensuu *et al.* (1994). Hellemans *et al.* (1995) did not observe a significant association between Bcl-2 and DFS or OS in 124 lymph node-negative breast cancer patients who did not receive adjuvant treatment.

Response to perioperative chemotherapy did not differ significantly among patients whose tumours expressed either undetectable to low or moderate to high levels of Bcl-2 (Figure 3c and 3d and Table III). These findings demonstrate that although moderate to high expression of Bcl-2 is a marker of favourable prognosis, Bcl-2 does not seem to predict response to adjuvant chemotherapy in this series of premenopausal, node-negative breast cancer patients. One may argue that only one cycle of perioperative chemotherapy cannot be considered an adequate treatment to assess the relationship between Bcl-2 expression and response to chemotherapy. However, the adjuvant chemotherapy regimen used in this trial can be described as a fairly intensive course of polychemotherapy. This regimen of perioperative chemotherapy resulted in a relatively small, but significant increase in DFS and OS in the group of premenopausal, lymph node-negative patients. Moreover, the benefit obtained with the single course of FAC used in this trial, was similar to results obtained with more prolonged administration of adjuvant chemotherapy in lymph node-negative patients (Early Breast Cancer Trialists' Collaborative Group, 1992). In this respect, it seems unlikely that the failure of Bcl-2 to predict response to chemotherapy in node-negative patients can be contributed to the treatment regimen used in this trial. In addition, it is important to note that the 'perioperative' chemotherapy, was given within 24 h after surgery and thus did not affect the expression level of Bcl-2.

An important finding, with respect to the association of high expression of Bcl-2 with improved survival, was the strong correlations observed between Bcl-2 expression and a number of important clinicopathological parameters (Figure 2a–f). High expression of Bcl-2 was positively correlated with well-differentiated tumours and low tumour grade and with ER and PgR positivity. High Bcl-2 expression was negatively correlated with p53 and c-erbB-2 positivity, with high MIB-1 (Ki-67) index and high mitotic index and with large tumour size. Thus, expression of Bcl-2 was associated with the presence of a number of classical prognostic factors known to predict a lower risk of relapse, providing a likely explanation for the improved survival of patients with tumours expressing high levels of Bcl-2.

An important finding, with regard to the failure of Bcl-2 to predict responsiveness, seemed to be the strong negative correlation between Bcl-2 expression and p53 protein accumulation, in concordance with recent reports by other groups (Leek *et al.*, 1994; Silvestrini *et al.*, 1994; Joensuu *et al.*, 1994; Gasparini *et al.*, 1995). Recently, evidence has been obtained that wild-type as well as most mutant p53 proteins can down-regulate Bcl-2 expression *in vitro* and *in vivo* (Haldar *et al.*, 1994; Selvakumaran *et al.*, 1994; Miyashita *et al.*, 1994). Mutations in the p53 tumour-suppressor gene are a marker of poor prognosis in node-negative breast cancer (Isola *et al.*, 1992) and experimental data have demonstrated increased resistance to doxorubicin and gamma irradiation in tumours lacking functional p53 (Lowe *et al.*, 1994). We also evaluated the predictive value of Bcl-2 expression on responsiveness in the subgroup of patients with p53-negative tumours. Again, Bcl-2 did not seem to predict response to chemotherapy. Thus, the failure of Bcl-2 to predict response to chemotherapy is not the result of the relatively high percentage of p53-positive tumours in the group of patients with negative to low expression of Bcl-2.

It should be realised that *bcl-2* is only one member of an expanding family of genes involved in cell death regulation, including *bcl-X* (Boise *et al.*, 1993), *mcl-1* (Reynolds *et al.*, 1994), *al* (Lin *et al.*, 1993), *bax* (Oltvai *et al.*, 1993), *bad* (Yang *et al.*, 1995) and *bak* (Kiefer *et al.*, 1995), that have not yet been studied in human cancer. The proteins encoded by these genes can bind to each other, forming homodimers and heterodimers, as well as physically interact with a number of other proteins. Co-expression of these proteins can either decrease or increase the cellular threshold for induction of apoptosis set by Bcl-2, and may explain the failure of Bcl-2 to predict responsiveness in this series of patients.

The observation that Bcl-2 is preferentially expressed in well-differentiated tumours is in line with data on Bcl-2 expression in other types of solid tumours (e.g. non-small cell lung cancer and thyroid carcinoma) (Pezzella *et al.*, 1993; Pilotti *et al.*, 1994). In thyroid carcinoma, *bcl-2* appeared to be under the control of differentiation-related transcription factors (Civitareale *et al.*, 1989). The data presented in this study suggest the presence of a similar differentiation-dependent regulation of *bcl-2* in breast epithelium. This hypothesis is supported by the strong positive correlations found between high Bcl-2 expression and ER postivity. Of interest, in this context, is that all observed cases of apocrine metaplasia were found to be completely negative for both Bcl-2 (Figure 1d) and ER (data not shown). These findings indicate that in breast epithelium Bcl-2 expression is under the control of oestrogen. Data on Bcl-2 expression in normal breast tissue and endometrium during the menstrual cycle support the hypothesis that *bcl-2* gene expression is hormonally regulated (Sabourin *et al.*, 1994; Gompel *et al.*, 1994).

A DCIS component was present in 65.5% of the cases and mostly showed a similar Bcl-2 expression as the invasive component (only 20.5% was judged to have a stronger or weaker staining intensity), suggesting that clonal variations in Bcl-2 expression are a relatively early event in tumour progression.

In conclusion, this study confirms that Bcl-2 is a strong, but not an independent marker of favourable prognosis and demonstrates that it has little predictive value for response to a single course of polychemotherapy in premenopausal, node-negative breast cancer patients.

### Abbreviations

BC, breast cancer; ER, oestrogen receptor; PgR, progesterone receptor; F, 5-fluorouracil; A, doxorubicin; C, cyclophosphamide; PBS, phosphate-buffered saline; BSA, bovine serum albumin; DFS, disease-free survival; OS, overall survival; SI, staining intensity; DCIS, ductal carcinoma *in situ*; HR, hazard ratio; CI, confidence interval; PeCT, perioperative chemotherapy; N, total number of patients; O, number of events.

### Acknowledgements

We thank Dr CJ Cornelisse (Department of Pathology, Leiden University Hospital, Leiden, The Netherlands) for his stimulating discussions and L van den Broek, R Keijzer, NJ Kuipers-Dijkshoorn and EJ Vink for their helpful assistance in the immunohistochemical staining of Bcl-2. This work was supported by the Dutch Cancer Society grant 91-03.

### References

BHARGAVA V, KELL DL, VAN DE RIJN M AND WARNKE RA. (1994). Bcl-2 immunoreactivity in breast carcinoma correlates with hormone receptor positivity. *Am. J. Pathol.*, **145**, 535–539.

BOISE LH, GONÁLEZ-GARCÍA M, POSTEMA CE, DING L, LINDSTEN T, TURKA LA, MAO X, NUNEZ G AND THOMPSON CB. (1993). *bcl-x, a bcl-2*-related gene that functions as a dominant regulator of apoptotic cell death. *Cell*, **74**, 597–608.

CAMPOS L, ROUAULT JP, SABIDO O, ORIOL P, ROUBI N, VASSELON C, ARCHIMBAUD E, MAGAUD JP AND GUYOTAT D. (1993). High expression of bcl-2 protein in acute myeloid leukemia cells is associated with poor response to chemotherapy. *Blood*, **81**, 3091–3096.

CASTLE VP, HEIDELBERGER KP, BROMBERG J, OU X, DOLE M AND NUNEZ G. (1993). Expression of the apoptosis-suppressing protein bcl-2, in neuroblastoma is associated with unfavorable histology and N-*myc* amplification. *Am. J. Pathol.*, **143**, 1543–1550.

CIVITAREALE D, LONIGRO R, SINCLAIR A AND DI LAURO R. (1989). A thyroid-specific nuclear protein essential for tissue-specific expression of the thyroglobulin promoter. *EMBO J.*, **8**, 2537–2542.

CLAHSEN PC, VAN DE VELDE CJH, DUVAL C, PALLUD C, MANDARD A-M, DELOBELLE-DEROIDE A, VAN DEN BROEK L, SAHMOUD T AND VAN DE VIJVER MJ. (1994*a*). Prognostic factors in premenopausal node-negative women with early breast cancer. *Eur. J. Cancer*, **30A** (suppl. 2), S18.

CLAHSEN PC, VAN DE VELDE CJH, JULIEN JP, FLOIRAS JL AND MIGNOLET FY. (1994*b*). Thromboembolic complications after perioperative chemotherapy in women with early breast cancer: A European Organization for Research and Treatment of Cancer Breast Cancer Cooperative Group Study. *J. Clin. Oncol.*, **12**, 1266–1271.

COX DR. (1972). Regression models and life-tables. *J. R. Stat. Assoc.*, **B34**, 187–220.

DE JONG D, PRINS F, MASON D, REED J, VAN OMMEN G AND KLUIN P. (1994). Subcellular localization of the bcl-2 protein in malignant and normal lymphoid cells. *Cancer Res.*, **54**, 256–260.

DEBBAS M AND WHITE E. (1993). Wild-type p53 mediates apoptosis by E1A, which is inhibited by E1B. *Genes Dev.*, **7**, 546–554.

EARLY BREAST CANCER TRIALISTS' COLLABORATIVE GROUP. (1992). Systemic treatment of early breast cancer by hormonal, cytotoxic, or immune therapy. *Lancet*, **339**, 1-15–71-85.

GASPARINI G, BARBARESCHI M, DOGLIONI C, DALLA PALMA P, MAURI FA, BORACCHI P, BEVILACQUA P, CAFFO O, MORELLI L, VERDERIO P, PEZZELLA F AND HARRIS AL. (1995). Expression of bcl-2 protein predicts efficacy of adjuvant treatments in operable node-positive breast cancer. *Clin. Cancer Res.*, **1**, 189–198.

GEE JM, ROBERTSON JF, ELLIS IO, WILLSHER P, MCCLELLAND RA, HOYLE HB, KYME SR, FINLAY P, BLAMEY RW AND NICHOLSON RI. (1994). Immunocytochemical localization of BCL-2 protein in human breast cancers and its relationship to a series of prognostic markers and response to endocrine therapy. *Int. J. Cancer.*, **59**, 619–628.

GOMPEL A, SABOURIN JC, MARTIN A, YANEVA H, AUDOUIN J, DECROIX Y AND POITOUT P. (1994). Bcl-2 expression in normal endometrium during the menstrual cycle. *Am. J. Pathol.*, **144**, 1195–1202.

HALDAR S, NEGRINI M, MONNE M, SABBIONI S AND CROCE CM. (1994). Down-regulation of bcl-2 by p53 in breast cancer cells. *Cancer Res.*, **54**, 2095–2097.

HELLEMANS P, VAN DAM PA, WEYLER J, VAN OOSTEROM AT, BUYTAERT P AND VAN MARCK E. (1995). Prognostic value of bcl-2 expression in invasive breast cancer. *Br. J. Cancer*, **72**, 354–360.

HOCKENBERY DM, ZUTTER M, HICKEY W, NAHM M AND KORSMEYER SJ. (1991). BCL2 protein is topographically restricted in tissue characterized by apoptotic cell death. *Proc. Natl Acad. Sci. USA*, **88**, 6961–6965.

ISOLA J, VISAKORPI T, HOLLI K AND KALLIONIEMI OP. (1992). Association of overexpression of tumor suppressor protein p53 with rapid cell proliferation and poor prognosis in node-negative breast cancer patients. *J. Natl Cancer Inst.*, **85(14)**, 1109–1114.

JOENSUU H, PYLKKANEN L AND TOIKKANEN S. (1994). Bcl-2 protein expression and long-term survival in breast cancer. *Am. J. Pathol.*, **145**, 1191–1198.

KAPLAN EL AND MEIER P. (1958). Non-parametric estimation from incomplete observations. *J. Am. Stat. Assoc.*, **53**, 457–481.

KIEFER MC, BRAUER MJ, POWERS VC, WU JJ, UMANSKY SR, TOMEL LD AND BARR PJ. (1995). Modulation of apoptosis by the widely distributed Bcl-2 homologue Bak. *Nature*, **374**, 736–739.

LEEK RD, KAKLAMANIS L, PEZZELLA F, GATTER KC AND HARRIS AL. (1994). bcl-2 in normal human breast and carcinoma, association with oestrogen receptor-positive, epidermal growth factor receptor-negative tumours and in situ cancer. *Br. J. Cancer.*, **69**, 135–139.

LIN EY, ORLOFSKY A, BERGER, MS AND PRYSTOWSKY MB. (1993). Characterization of A1, a novel hemopoietic-specific early-response gene with sequence similarity to bcl-2. *J. Immunol.*, **151**, 1979–1988.

LOWE SW, SCHMITT EM, SMITH SW, OSBORNE BA AND JACKS T. (1993). p53 is required for radiation-induced apoptosis in mouse thymocytes (see comments). *Nature*, **362(6423)**, 847–849.

LOWE SW, BODIS S, MCCLATCHEY A, REMINGTON L, RULEY HE, FISHER DE, HOUSMAN DE AND JACKS T. (1994). p53 status and the efficacy of cancer therapy in vivo. *Science*, **266**, 807–810.

MANTEL N. (1966). Evaluation of survival data and two new rank order statistics arising in its consideration. *Cancer Chemother. Rep.*, **50**, 163–170.

MERRITT AJ, POTTEN CS, KEMP CJ, HICKMAN JA, BALMAIN A, LANE DP AND HALL PA. (1994). The role of p53 in spontaneous and radiation-induced apoptosis in the gastrointestinal tract of normal and p53-deficient mice. *Cancer Res.*, **54**, 614–617.

MIYASHITA T, HARIGAI M, HANADA M AND REED JC. (1994). Identification of a p53-dependent negative response element in the *bcl-2* Gene. *Cancer Res.*, **54**, 3131–3135.

OLTVAI ZN, MILLIMAN CL AND KORSMEYER SJ. (1993). Bcl-2 heterodimerizes in vivo with a conserved homolog, Bax, that accelerates programmed cell death. *Cell*, **74**, 609–619.

PEZZELLA F, TURLEY H, KUZU I, TUNGEKAR MF, DUNNILL MS, PIERCE CB, HARRIS A, GATTER KC AND MASON DY. (1993). Bcl-2 protein in non-small-cell lung carcinoma. *N. Engl. J. Med.*, **329**, 690–694.

PILOTTI S, COLLINI P, RILKE F, CATTORETTI G, DEL BO R AND PIEROTTI MA. (1994). bcl-2 protein expression in carcinomas originating from the follicular epithelium of the thyroid gland. *J. Pathol.*, **172**, 337–342.

REED JC. (1994). Bcl-2 and the regulation of programmed cell death. (Review). *J. Cell Biol.*, **124**, 1–6.

REYNOLDS JE, YANG T, QIAN L, JENKINSON JD, ZHOU P, EASTMAN A AND CRAIG RW. (1994). Mcl-1, a member of the Bcl-2 family, delays apoptosis induced by c-myc overexpression in chinese hamster ovary cells. *Cancer Res.*, **54,** 6438-6352.

SABOURIN JC, MARTIN A, BARUCH J, TRUC JB, GOMPEL A AND POITOUT P. (1994). bcl-2 expression in normal breast tissue during the menstrual cycle. *Int. J. Cancer*, **59,** 1-6.

SELVAKUMARAN M, LIN H.-K, MIYASHITA T, WANG HG, KRAJEWSKI S, REED JC, HOFFMAN B AND LIEBERMANN D. (1994). Immediate early up-reulation of bax expression by p53 but not TGF-B1: a paradigm for distinct apoptotic pathways. *Oncogene*, **9,** 1791-1798.

SILVESTRINI R, VENERONI S, DAIDONE MG, BENINI E, BORACCHI P, MEZZETTI M, DI FRONZO G, RILKE F. AND VERONESI U. (1994). The bcl-2 protein: A prognostic indicator strongly related to p53 protein in lymph node-negative breast cancer patients. *J. Natl Cancer Inst.*, **86,** 499-504.

VAN DE VIJVER MJ, PETERSE JL, MOOL WJ, *et al.* (1988). Neu-protein overexpression in breast cancer. Association with comedo-type ductal carcinoma in situ and limited prognostic value in stage II breast cancer. *N. Engl. J. Med.*, **319**, 1239-1245.

YANG E, ZHA J, JOCKEL J, BOISE LH, THOMPSON CB AND KORSMEYER SJ. (1995). Bad, a heterodimeric partner for Bcl-$X_L$ and Bcl-2, displaces Bax and promotes cell death. *Cell*, **80,** 285-291.

YIN XM; OLTVAI ZN AND KORSMEYER SJ. (1995). Heterodimerization with Bax is required for Bcl-2 to repress cell death. *Curr. Top. Microbiol. Immunol.*, **194,** 331-338.

ZHU Y.-M, BRADBURY DA AND RUSSELL NH. (1994). Wild-type p53 is required for apoptosis induced by growth factor deprivation in factor-dependent leukaemic cells. *Br. J. Cancer*, **69,** 468-472.